# Outcome and prognostic factors of CBF pediatric AML patients with t(8;21) differ from patients with inv(16)

**DOI:** 10.1186/s12885-023-10965-5

**Published:** 2023-05-25

**Authors:** Kun-yin Qiu, Xiong-yu Liao, Yang Li, Ke Huang, Hong-gui Xu, Jian-pei Fang, Dun-hua Zhou

**Affiliations:** 1 Department of Hematology/Oncology, Children’s Medical Center, SunYat-Sen Memorial Hospital, Sun Yat-Sen University, Guangzhou, 510120 P. R. China; 2 Guangdong Provincial Key Laboratory of Malignant Tumor Epigenetics and Gene Regulation, Sun Yat-Sen Memorial Hospital, Sun Yat-Sen University, Guangzhou, 510120 P. R. China

**Keywords:** AML, t(8;21), inv(16), Outcome, Prognostic factors

## Abstract

**Purpose:**

To explore the outcome and prognostic factors between inv(16) and t(8;21) disrupt core binding factor (CBF) in acute myeloid leukemia (AML).

**Methods:**

The clinical characteristic, probability of achieving complete remission (CR), overall survival (OS) and cumulative incidence of relapse (CIR) were compared between inv(16) and (8;21).

**Results:**

The CR rate was 95.2%, 10-year OS was 84.4% and CIR was 29.4%. Subgroup analysis showed that patients with t(8;21) had significant lower 10-year OS and CIR than patients with inv(16). Unexpectedly, there was a trend for pediatric AML receiving five courses cytarabine to have a lower CIR than four courses cytarabine (19.8% vs 29.3%, *P* = 0.06). Among the cohort of no-gemtuzumab ozogamicin(GO) treatment, inv (16) patients showed a similar 10-year OS (78.9% vs 83.5%; *P* = 0.69) and an inferior outcome on 10-year CIR (58.6% vs 28.9%, *P* = 0.01) than those patients with t(8;21). In contrast, inv (16) and t(8;21) patients receiving GO treatment had comparable OS (OS: 90.5% vs. 86.5%, *P* = 0.66) as well as CIR (40.4% vs. 21.4%, *P* = 0.13).

**Conclusion:**

Our data demonstrated that more cumulative cytarabine exposure could improve the outcome of childhood patients with t(8;21), while GO treatment was beneficial to the pediatric patients with inv(16).

**Supplementary Information:**

The online version contains supplementary material available at 10.1186/s12885-023-10965-5.

## Introduction

The cytogenetic abnormalities inv (16)(p13.1q22)/t(16;16)(p13.1;q22)[hereafter referred to as inv (16)] and t(8;21)(q22;q22), commonly referred to as core binding factor (CBF) acute myeloid leukemia (AML) [1]. CBF-AML accounts for approximately 15% of AML in adults and slightly more than adults in children, accounting for 25% to 30% [2]. Although CBF-AML children were sensitive to chemotherapy, with a complete remission (CR) rate of 90% and a relatively high overall survival (OS) in the range of 85%, some children still experience relapse [3].

These two cytogenetic subgroups (collectively referred to as CBF-AML) have also been associated with a relatively favorable prognosis compared with patients with normal or adverse karyotypes, and clinical studies have often stratified these patients together, into one favorable-risk prognostic factor, and treated them similarly [4]. In recent years, a growing number of studies have shown that these two subgroups are highly heterogeneous and it remains controversial whether they should be treated equally. As most reports currently focus on children and adults together, independent reports on large samples of pediatric CBF-AML are rare [2–4].

In this present study, our purpose was to compared the clinical characteristic and prognostic factors on long-term outcome of 176 childhood patients with inv(16) AML with those of 251 pediatric patients with t(8;21) AML from the Therapeutically Applicable Research to Generate Effective Treatments (TARGET) database.

## Patients and methods

### Study participant

Finally, from September 2006 to December 2017, 427 consecutive children aged 0–18 years, newly diagnosed with AML, bone marrow (BM) and/or blood cytogenetic analysis was successful, and inv (16) or t (8; 21) were included in the TARGET data set. The results published in this paper are based in whole or in part on the data generated in the Research on Therapeutic Application to Produce Effective Treatment (https://ocg.cancer.gov/programs/target) Initiative, phs00218. The data used for this analysis can be found in the https://portal.gdc.cancer.gov/projects. The study was approved by the ethics committee of Sun Yat-sen Memorial Hospital, Sun Yat-sen University. The guardians of the patients signed the informed consent form. Our research is based on the Helsinki Declaration. According to TARGET data, the enrollment year was 2006 to 2017, and the last follow-up year was 2008 to 2019.

### Treatment protocol

Children with CBF-AML were treated with AAML1031 or AAML0531. In AAML1031 (Supplementary Table 1), low-risk (LR) patients received four courses of chemotherapy, including two induction courses of cytarabine/daunorubicin/etoposide and two consolidation courses: cytarabine and etoposide, followed by cytarabine or mitoxantrone. In AAML0531 (Supplementary Table 2), LR patients plan to receive five courses of chemotherapy, including the same four courses as AAML1031, and an additional fifth course consisting of high-dose cytarabine/L-asparaginase. Four courses of treatment are equivalent to about half of the accumulated cytarabine exposure of five courses of treatment (i.e. 21.6 g/m^2^ vs 45.6 g/m^2^), and there is no difference in anthracycline drug exposure.

### Definition of clinical outcome

Complete remission (CR) was defined as restoration of normal bone marrow (BM) and normal blood cell count (i.e. neutrophils ≥ 1.5 × 10^9^/L, platelet ≥ 100 × 10^9^/L), no evidence of circulating leukemia mother cells or extramedullary leukemia. Relapse was defined as the development of ≥ 5% myeloblasts, circulating leukemic blasts, or extramedullary leukemia. The overall survival rate (OS) was measured from the study entry until the death date or the last survival date. The cumulative incidence of recurrence (CIR) was measured only in patients who received CR, from the date of CR to the date of relapse, the date of death, or the last known date of survival, where CR death was considered a competitive risk.

### Statistical analysis

Fisher exact and Wilcoxon rank sum tests compare categorical variables and continuous variables, respectively. Multivariate cox proportional risk model was used to determine the independent influence of prognostic factors on OS and CIR in AML patients. Kaplan Meier method was used to calculate the estimated probability of OS, and log-rank test was used to evaluate the difference between survival curves. The estimated value of CIR was calculated, and Grey's k-sample test was used to evaluate the difference of relapse rate.SPSS statistical software version 22.0 and EmpowerStats were used for all statistical analysis (http://www.empowerstats.cn/). *P* < 0.05 was considered statistically significant.

## Results

### Baseline clinical characteristics of CBF-AML childhood patients

Of 427 childhood patients, 176 had inv(16) and 251 had t(8;21). Among them, 218(51.1%) were male and 209(48.9%) were female, and the median age in the whole cohort was 10.9 years old. The two groups differed significantly in several characteristics (Table 1). White race were more common in inv(16) when compared with t(8;21) (85% vs 71.2%, *P* = 0.002). Patients with t(8;21) were more likely to have FAB M2 phenotype while FAB M4 were more frequent in inv(16). The initial median WBC of patients with inv(16) was much higher than that of t(8;21) (66.5 × 10^9^/L vs 16.5 × 10^9^/L, *P* < 0.001). Chloroma were less common in inv(16) when compared with t(8;21) (8.3% vs 16.2%, *P* = 0.045). BM relapse and central nervous system (CNS) relapse among children with inv(16) were significant higher than those with t(8;21) (BM relapse: 31.2% vs 23%, *P* = 0.049; CNS relapse: 11.1% vs 2.3%, *P* < 0.001). There was a trend towards a lower proportion of CNSL in patients with inv(16) than those with t(8;21) (52.3% vs 61.3%; *P* = 0.066).

**Table 1:** Baseline clinical characteristics of CBF AML patients

Characteristics	Total (*n* = 427)	inv (16) (*n* = 176)	t (8; 21) (*n* = 251)	*P* value
Gender, n(%)				0.715
Male	218 (51.1%)	88 (50.0%)	130 (51.8%)	
Female	209 (48.9%)	88 (50.0%)	121 (48.2%)	
Age(y), median(range)	10.9 (0.3–17.9)	10.6 (0.3–17.9)	11.1 (0.6–17.8)	0.059
Race				0.002
White	289 (77.1%)	136 (85.0%)	153 (71.2%)	
Nonwhite	86 (22.9%)	24 (15.0%)	62 (28.8%)	
Ethnicity				0.177
Hispanic or Latino	96 (23.4%)	34 (20.0%)	62 (25.7%)	
Not Hispanic or Latino	315 (76.6%)	136 (80.0%)	179 (74.3%)	
FAB Category				< 0.001
M1	12 (6.8%)	0 (0.0%)	12 (11.8%)	
M2	88 (49.7%)	0 (0.0%)	88 (86.3%)	
M4	75 (42.4%)	74 (98.7%)	1 (1.0%)	
M5	2 (1.1%)	1 (1.3%)	1 (1.0%)	
Chemotherapy protocol, n (%)				0.387
AAML1031	229 (53.6%)	90 (51.1%)	139 (55.4%)	
AAML0531	198 (46.4%)	86 (48.9%)	112 (44.6%)	
Initial WBC (× 10^9^/L), median (range)	30.8 (0.6–478.5)	66.5 (2.2–478.5)	16.5 (0.6–309.3)	< 0.001
PB blast(%)	42.0 (0.0–98.0)	43.4 (0.0–94.0)	42.0 (0.0–98.0)	0.730
BM blast (%)	59.0 (0.0–100.0)	61.5 (6.0–100.0	57.0 (0.0–99.0)	0.166
Risk group, n(%)				0.703
Low risk	419 (98.6%)	172 (98.9%)	247 (98.4%)	
High risk	6 (1.4%)	2 (1.1%)	4 (1.6%)	
CNSL, n(%)				0.066
No	179 (42.4%)	83 (47.7%)	96 (38.7%)	
Yes	243 (57.6%)	91 (52.3%)	152 (61.3%)	
Chloroma				0.045
No	261 (87.0%)	111 (91.7%)	150 (83.8%)	
Yes	39 (13.0%)	10 (8.3%)	29 (16.2%)	
CR status at end of course 1				0.062
CR	353 (84.2%)	149 (86.6%)	204 (82.6%)	
Not in CR	59 (14.1%)	18 (10.5%)	41 (16.6%)	
Death	7 (1.7%)	5 (2.9%)	2 (0.8%)	
CR status at end of course 2				0.441
CR	394 (95.2%)	158 (94.0%)	236 (95.9%)	
Not in CR	8 (1.9%)	5 (3.0%)	3 (1.2%)	
Death	12 (2.9%)	5 (3.0%)	7 (2.8%)	
GO treatment				0.586
No	56 (50.9%)	21 (47.7%)	35 (53.0%)	
Yes	54 (49.1%)	23 (52.3%)	31 (47.0%)	
Bone Marrow Site of Relapse				0.049
No	341 (73.5%)	137 (68.8%)	204 (77.0%)	
Yes	123 (26.5%)	62 (31.2%)	61 (23.0%)	
CNS Site of Relapse				< 0.001
No	436 (94.0%)	177 (88.9%	259 (97.7%)	
Yes	28 (6.0%)	22 (11.1%)	6 (2.3%)	
Other Site of Relapse				0.704
No	458 (98.7%)	197 (99.0%)	261 (98.5%)	
Yes	6 (1.3%)	2 (1.0%)	4 (1.5%)	

*Abbreviations*: *WBC* white blood cell, *PB* peripheral blood, *BM* bone marrow, *CNSL* central nervous system leukemia, *CR* complete remission, *GO* Gemtuzumab ozogamicin

### Secondary cytogenetic abnormalities among patients with CBF-AML

Table 2 summarized the most common secondary cytogenetic abnormalities in each cytogenetic group. The prevalence of secondary cytogenetic abnormalities in the whole cohort was 43.2%. As shown in Table 2, del (9q) were only found in pediatric AML with t(8;21) by a percentage of 16.6%. Similar results also appeared in minus X between inv(16) and t(8;21) (19.6% vs 0, *P* < 0.001). Within the cohort with minus Y group, patients with inv(16) had significant lower prevalence than patients with t(8;21) (0.5% versus 30.2%; *P* < 0.001). There was a trend towards a higher proportion of trisomy 8 in patients with inv(16) than those with t(8;21) (9.1% vs 4.9%; *P* = 0.072). No significant difference were observed in FLT3/ITD, NPM1, WT1 and CEBPA mutation. Of these 427 patients, 205 had assessable samples for c-kit mutational analysis. Analysis included PCR amplification of exons 8 and 17 and fragment length analysis and direct sequencing to identify all missense and size mutations. Mutations were detected in 52 patient samples (25.4%); 28 (53.8%) patients involved exon 8, 22 (42.3%) patients involved exon 17 and 2 (3.9%) patients involved both exons. When restricted to CBF translocation type, we found exon 8 mutations in 17.2% inv(16) samples and 11.5% t(8;21) patient samples. Exon 17 mutations were observed in 10.8% inv(16) and 14.3% t(8;21) patient samples.

**Table 2:** Secondary cytogenetic abnormalities among patients with CBF AML

Secondary Cytogenetic Abnormalities	inv (16)		t (8; 21)		*P* value
	No	%	No	%	
del(7q)					0.110
No	182	92.4	254	95.8	
Yes	15	7.6	11	4.2	
del(9q)					< 0.001
No	197	100	221	83.4	
Yes	0	0	44	16.6	
Trisomy 8					0.072
No	179	90.9	252	95.1	
Yes	18	9.1	13	4.9	
Minus X					< 0.001
No	197	100	213	80.4	
Yes	0	0	52	19.6	
Minus Y					< 0.001
No	196	99.5	185	69.8	
Yes	1	0.5	80	30.2	
Complex Cytogenetic					< 0.001
1	121	61.4	66	24.9	
2	51	25.9	133	50.2	
> 3	25	12.7	66	24.9	
KMT2A status					0.246
Negative	196	99.5	265	100	
Positive	1	0.5	0	0	
FLT3-ITD Status					0.660
Negative	189	95	253	95.8	
Positive	10	5	11	4.2	
NPM1 Status					0.249
Negative	198	99.5	264	100	
Positive	1	0.5	0	0	
CEBPA Status					0.843
Negative	198	99.5	262	99.6	
Positive	1	0.5	1	0.4	
WT1 Status					0.296
Negative	92	95.8	112	98.2	
Positive	4	4.2	2	1.8	
c-Kit Mutation Exon 8					0.242
Negative	77	82.8	100	88.5	
Positive	16	17.2	13	11.5	
c-Kit Mutation Exon 17					0.449
Negative	83	89.2	96	85.7	
Positive	10	10.8	16	14.3	
c-Kit Mutation					0.649
Negative	68	73.1	85	75.9	
Positive	25	26.9	27	24.1	

### Clinical outcome of CBF-AML pediatric patients

**Fig. 1:**
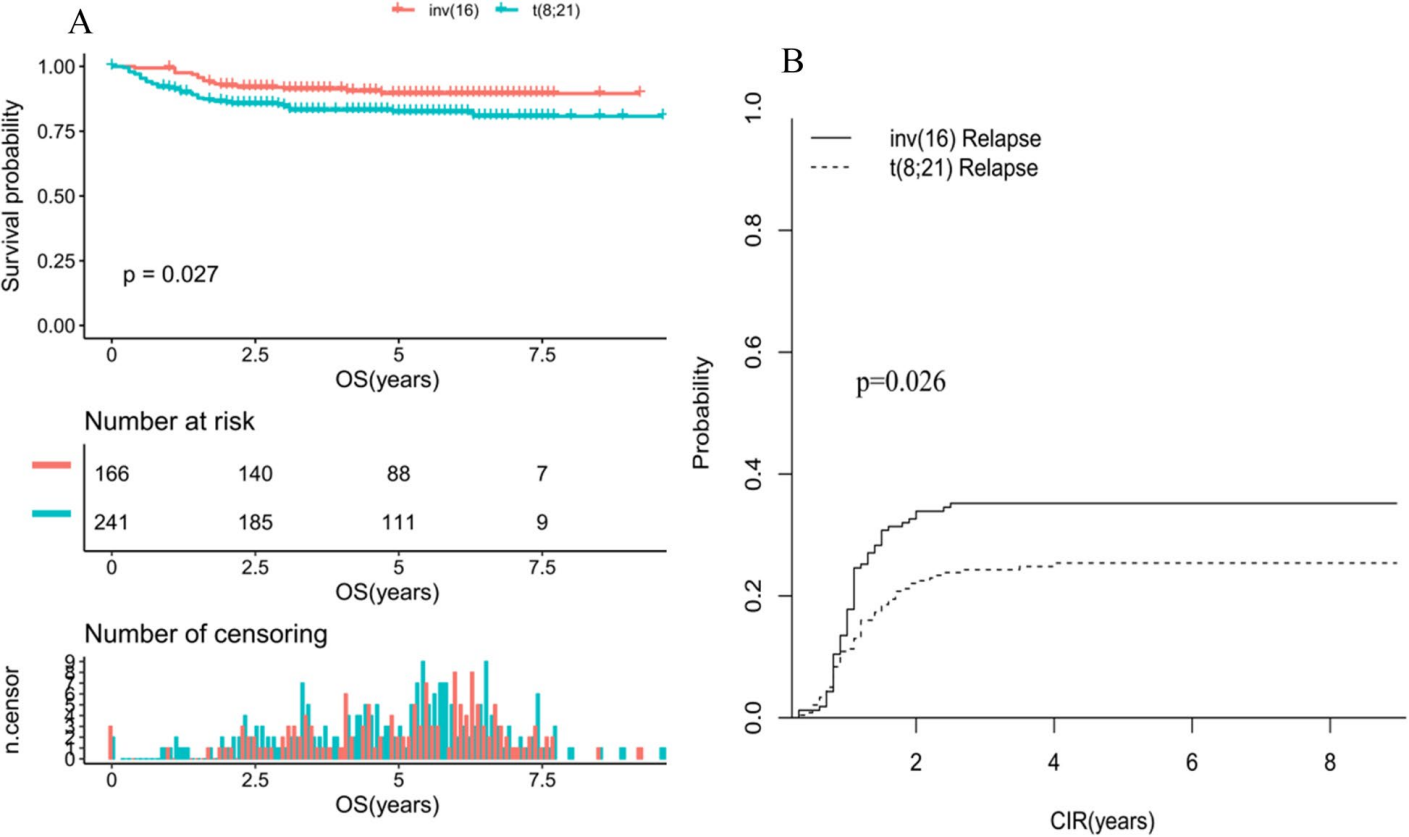
**A** Comparison of OS in patients with inv(16) and t(8;21) AML (**B**) Comparison of CIR in patients with inv(16) and t(8;21) AML

Of the 427 patients who received either AAML1031 or AAML0531 were for evaluable response, the total CR of the whole CBF-AML pediatric patients were 95.2%, and 94% with inv(16) and 95.9% with t(8; 21) achieved a CR (*P* = 0.441), respectively (Table 3). Patients with t(8;21) showed a significant shorter OS compared with patients with inv(16) (80.7% vs 89.5%, *P* = 0.027; Fig. 1A). Notably, t(8;21) patients who achieved CR had a lower CIR of 25.4% vs. 35.2% for inv(16) patients (*P* = 0.026; Fig. 1B). Most importantly, in the multivariate analysis, FLT3-ITD positive (*P* = 0.019) and c-Kit Mutation (*P* = 0.005) were the independent factors that can adversely affect CIR when the two cytogenetic groups were considered together (Table 4). In addition to having inv(16), not Hispanic or Latino (*P* = 0.02), FLT3-ITD positive (*P* = 0.05) and c-Kit mutation (*P* = 0.04) were the independent factors which significantly increased the risk of relapse (Table 4), while gemtuzumab ozogamicin (GO) treatment (*P* = 0.02) were significantly associated with a decreased relapse, whereas no significant prognostic factors were identified for t(8;21) in terms of CIR.

**Table 3:** Outcome of the pediatric CBF AML patient population

	CBF		inv(16)		t(8;21)		*P*
	%	95%CI	%	95%CI	%	95%CI	
CR	95.2		94		95.9		0.441
CIR							
Median, years	3.9		3.6		4.1		
10 years	29.4	24.9–33.9	35.2	27.9–42.6	25.4	19.9–31.2	0.026
OS							
Median, years	4.9		5.2		4.6		
10 years	84.4	80.5–88.5	89.5	84.7–94.6	80.7	75.1–86.8	0.027

**Table 4:** Multivariable models of outcome for patients with pediatric CBF AML

Outcome	Variable	CBF		inv(16)		t(8; 21)	
		HR (95% CI)	*P*	HR (95% CI)	*P*	HR (95% CI)	*P*
CIR	Not Hispanic or Latino	1.0 (0.4, 2.8)	0.966	16 (1.7–151.8)	0.02	0.3 (0.1, 1.3)	0.095
	FLT3-ITD positive	4.5 (1.3, 15.6)	0.019	4.2 (1.0–18.3)	0.05	10.9 (0.7, 178.8)	0.095
	GO treatment	0.7 (0.3, 1.4)	0.290	0.3 (0.1–0.8)	0.02	1.0 (0.3, 3.7)	0.990
	c-Kit Mutation	2.2 (1.3–3.9)	0.005	6.1 (1.8–20.6)	0.04	NA	NA
OS	Not Hispanic or Latino	0.2 (0.1–0.7)	0.01	NA	NA	0.1 (0.0, 0.6)	0.01
	FLT3-ITD positive	21.6 (4.1–114.9)	< 0.001	25.9 (3.9–169.3)	< 0.001	NA	NA
	c-Kit Mutation Exon 8	0.1 (0–0.7)	0.02	NA	NA	NA	NA
	c-Kit Mutation	5.8 (1.7–19.8)	0.01	0.6 (0.0, 10.7)	0.708	6.2 (1.4–27.5)	0.02
	Nonwhite	0.2 (0.1–1.1)	0.06	0.2 (0.0, 8.2)	0.390	0.3 (0.1–1.8)	0.18
	Secondary Chromosome Abnormalities	1.9 (0.5, 7.4)	0.36	NA	NA	3.7 (0.7–20.1)	0.13

The favorable impact of the whole cohort on OS were non-Hispanic or Latino (*P* = 0.01) and c-Kit mutation exon 8 (*P* = 0.02), and nonwhite race had a moderate interaction (*P* = 0.06). When the analysis was restricted to patients with inv(16), FLT3-ITD positive showed a strong association with a lower survival (*P* < 0.001). Among t(8;21) cohort, not Hispanic or Latino had a significant better survival (*P* = 0.01), whereas c-kit mutation associated with a lower survival rate (*P* = 0.02), and a moderate interaction was observed between nonwhite race (*P* = 0.18) and secondary chromosome abnormalities (*P* = 0.13).

### Outcome of LR population by number of treatment courses

In the current study, we sought to determine whether the benefit from more cytarabine exposure was similar in LR patients with inv(16) and those with t(8;21). After excluding high-risk patients (*n* = 6) and death during induction (*n* = 19, 4 with inv 16 and 15 with t(8:21)), 402 LR patients were eventually included in the analysis. The subsequent analyses comparing five and four course cytarabine exposure were conducted for the whole CBF-AML patients and then for the inv(16) and t(8;21) groups, respectively. Patients in the two cytogenetic groups, five course (*n* = 218) and four courses (*n* = 184), had similar presenting characteristic at initial diagnosis (data not shown). The estimated 10-year rates of OS and CIR were summarized in Table 5.

**Table 5:** Outcome of the low-risk CBF AML study population by number of treatment courses received

	Four courses (*n* = 218)		Five courses (*n* = 184)		*P* value
	%	95%CI	%	95%CI	
CBF (*n* = 402)					
10-years CIR	30.9	24.7–37.2	27.4	21.2–34.4	0.42
10-years OS	82.8	75.1–91.2	85.4	80.4–90.8	0.91
inv(16) (*n* = 164), No. of patients	83		81		
10-years CIR	33.9	23.7–44.5	36.1	25.7–46.6	0.67
10-years OS	92.2	86.5–98.4	88.4	81.5–95.8	0.52
t(8;21) (*n* = 238), No. of patients	135		103		
10-years CIR	29.3	21.7–37.3	19.8	12.6–28.1	0.06
10-years OS	77	65.8–90.2	83	75.9–90.7	0.73

**Fig. 2:**
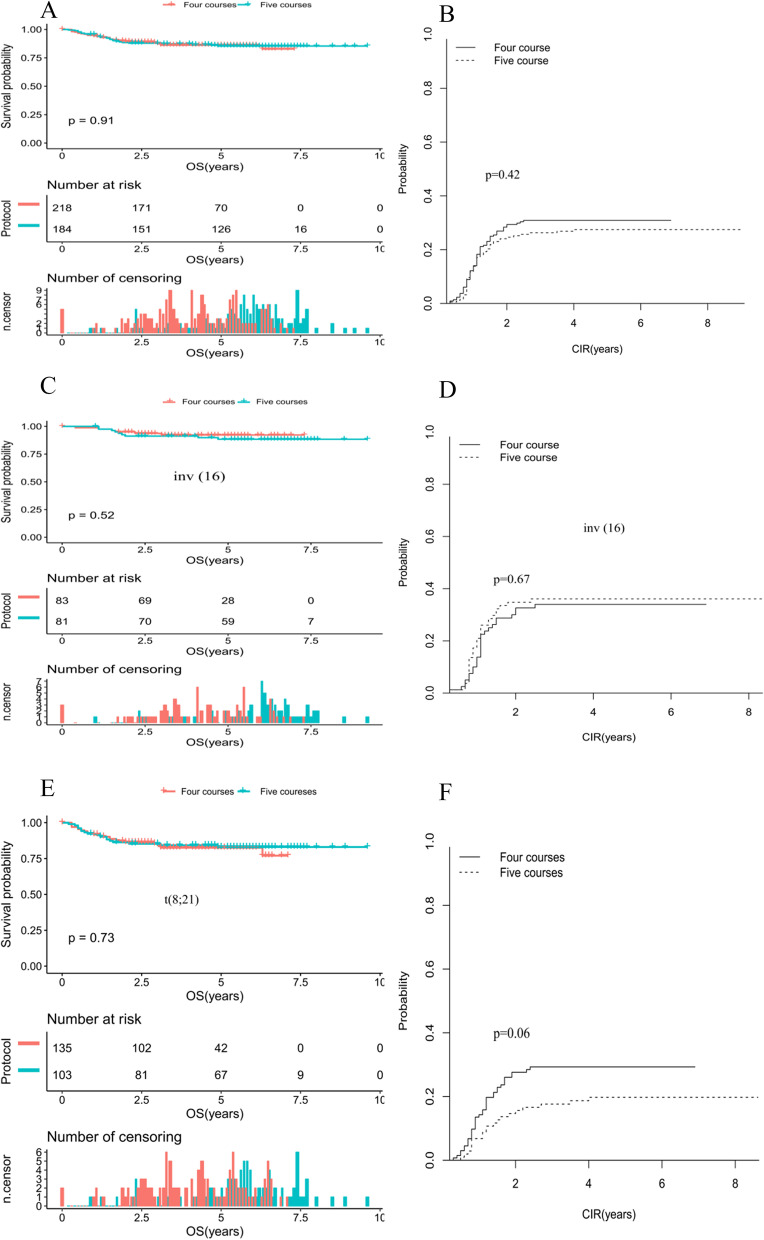
**A** Comparison of OS in patients with CBF-AML according chemotherapy courses. **B** Comparison of CIR in patients with CBF-AML according chemotherapy courses. **C** Comparison of OS in patients with inv(16) according chemotherapy courses. **D** Comparison of CIR in patients with inv(16) according chemotherapy courses (E)Comparison of OS in patients with t(8;21) according chemotherapy course. **F** Comparison of CIR in patients with t(8;21) according chemotherapy course

Similar difference was observed between four and five courses in terms of OS (82.8% vs. 85.4%, *P* = 0.91; Fig. 2A), which was not statistically significant. Patients with CBF assigned to either four or five courses seemed to have comparable risk of relapse (30.9% vs 27.4%, *P* = 0.42; Fig. 2B). Among inv(16) population, the patients who received five courses were slightly higher than those with four courses as regards OS and CIR(Fig. 2C-D). Whereas restricted to the t(8;21) cohort, the children received the four courses resulted in a relative lower OS compared to those received five courses (77% vs 83%, *P* = 0.73; Fig. 2E), but without any significant differences. Interestingly, there was a trend for pediatric AML (19.8% vs 29.3%, *P* = 0.06; Fig. 2F).

### The impact of c-kit mutation and GO treatment for pediatric CBF-AML

The impact of GO treatment on outcome was subsequently evaluated for pediatric CBF-AML patients. According to AAML0531 protocol, childhood patients randomized to receive GO treatment while AAML1031 protocol did not include any additional GO treatment. The estimated 10-year rates of OS and CIR were summarized in Table 6.

**Table 6:** Outcome of the low-risk CBF AML study population by GO treatment received

Outcome	CBF		inv(16)		t(8; 21)		*P* value
	%	95% CI	%	95% CI	%	95% CI	
GO (*n* = 52)							
CIR							
Median, years	5.1		3.2		5.6		
10 years	28.1	14.9–39.2	40.4	16–57.7	21.4	4.2–32.2	0.13
OS							
Median, years	5.8		5.4		5.8		
10 years	88.6	80.4–97.6	90.5	78.8–100	86.5	75.1–99.7	0.66
No-GO (*n* = 51)							
CIR							
Median, years	4.8		1.1		5.2		
10 years	39.8	26.2–53.1	58.6	32.3–77.6	28.9	14.2–45.4	0.01
OS							
Median, years	5.4		5.8		5.4		
10 years	80.1	69.8–91.9	78.9	62.6–99.6	83.5	71.2–97.8	0.69

**Fig. 3:**
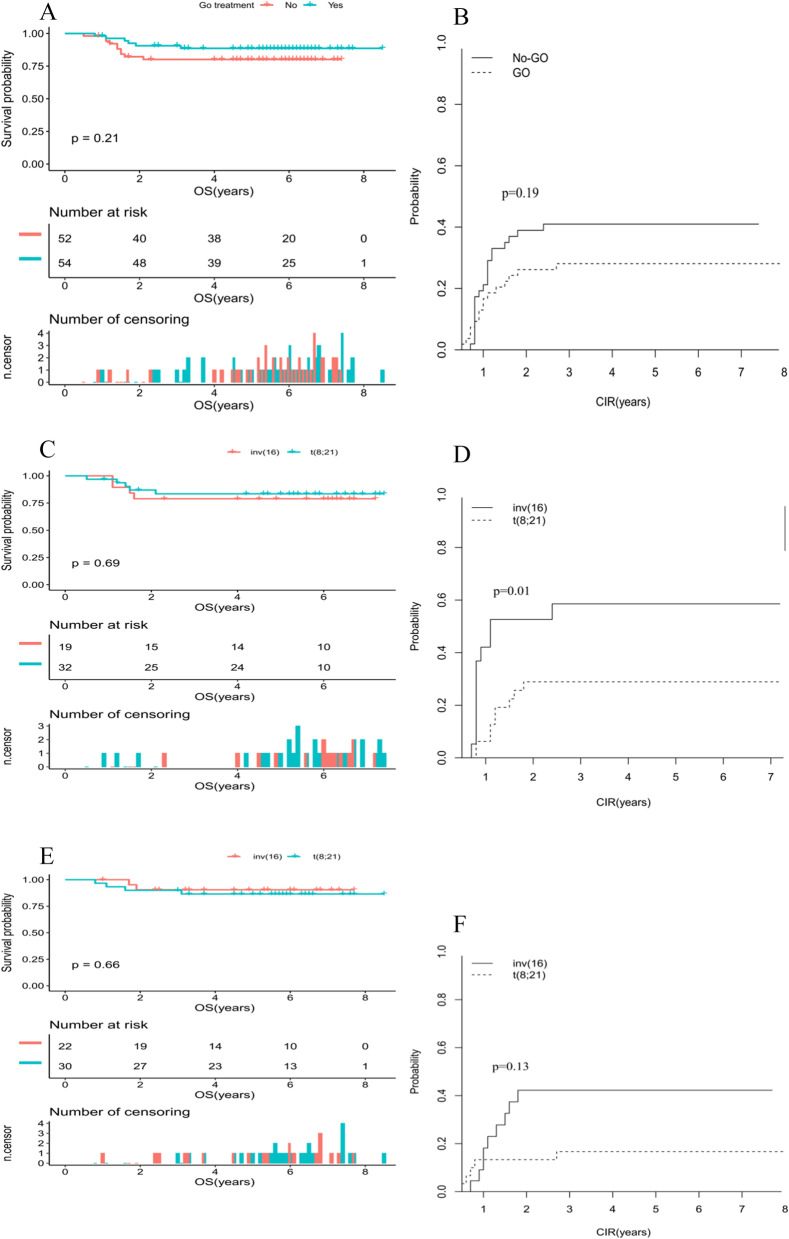
**A** Comparison of OS in patients with CBF-AML according GO treatment. **B** Comparison of CIR in patients with CBF-AML according GO treatment. **C** Comparison of OS in patients with inv(16) and t(8; 21) among the No-GO treatment cohort. **D** Comparison of CIR in patients with inv(16) and t(8; 21) among the No-GO treatment cohort. **E** Comparison of OS in patients with inv(16) and t(8; 21) among the GO treatment cohort. **F** Comparison of CIR in patients with inv(16) and t(8; 21) among the GO treatment cohort

Corresponding OS at 10 years for those with GO and No-GO was 88.6% and 80.1% (*P* = 0.12; Fig. 3A). CIR at 10 years for patients with GO and No-GO was 28.1% and 39.8% (*P* = 0.19; Fig. 3B). Within the cohort of No-GO treatment, inv (16) patients showed a similar 10-year OS (78.9% vs 83.5%; *P* = 0.69 l; Fig. 3C) and an inferior outcome on 10-year CIR (58.6% vs 28.9%, *P* = 0.01; Fig. 3D) than those patients with t(8;21). In contrast, inv (16) and t(8;21) patients receiving GO treatment had comparable OS (OS: 90.5% vs. 86.5%, *P* = 0.66; Fig. 3E) as well as CIR (40.4% vs. 21.4%, *P* = 0.13; Fig. 3F).

**Fig. 4:**
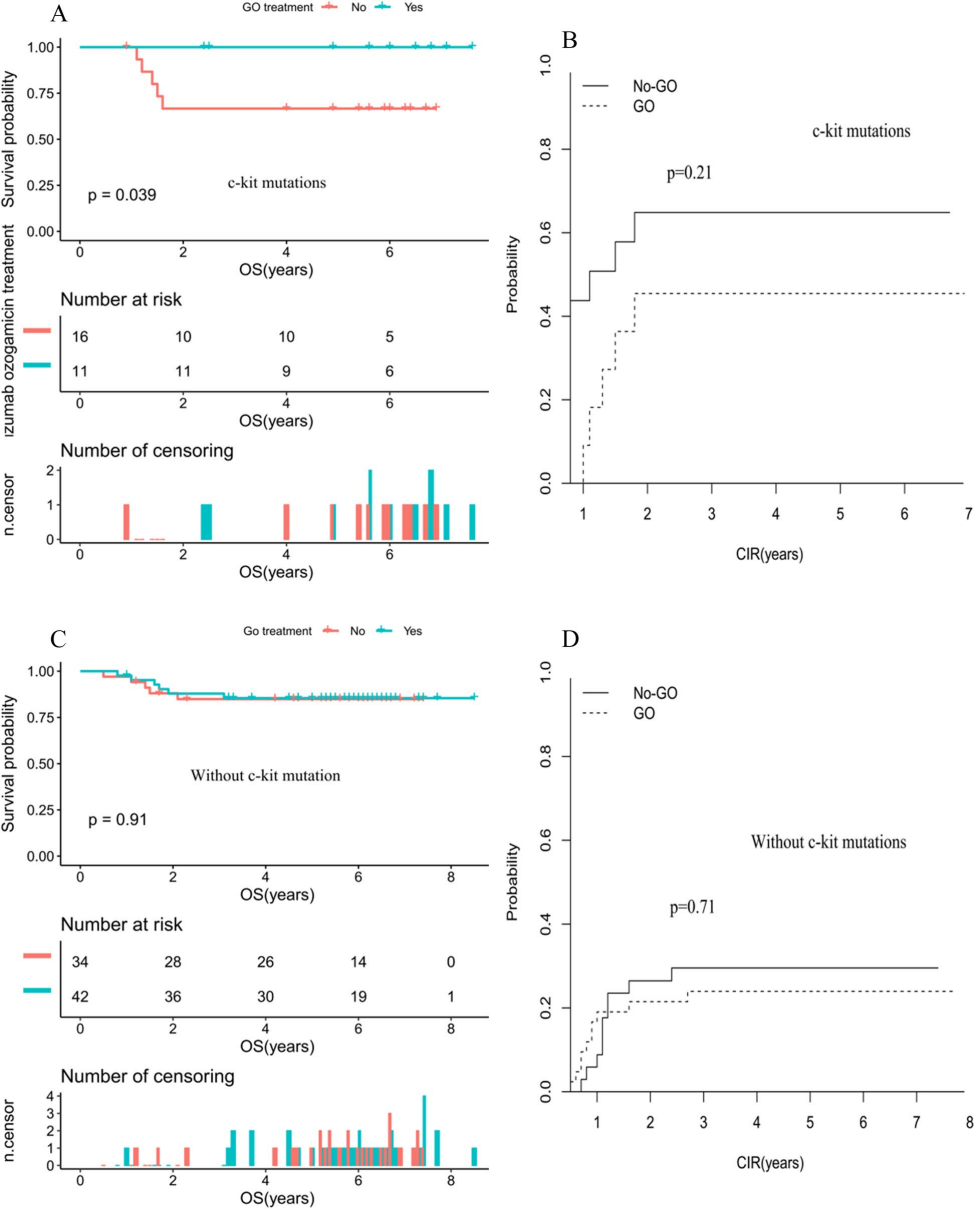
**A** Comparison of OS in patients with c-kit mutations according GO treatment. **B** Comparison of CIR in patients with c-kit mutations according GO treatment. **C** Comparison of OS in patients without c-kit mutations according GO treatment. D Comparison of CIR in patients without c-kit mutations according GO treatment

Importantly, for patients with c-kit mutations, treatment with GO treatment resulted in superior outcomes when compared to those without GO treatment (OS: 100% vs. 66.7%, *P* = 0.039, Fig. 4A; CIR: 45.5% vs. 64.9%, *P* = 0.21, Fig. 4B). Whereas in patients without c-kit mutations, no impact were found between GO and No-GO treatment group (OS: 100% vs. 66.7%, *P* = 0.039; CIR: 45.5% vs. 64.9%, *P* = 0.21) (Fig. 4C, D).

## Discussion

In this study, it was found that patients with inv (16) were far common in White race than those with t(8; 21), which indicated that the prevalence of CBF-AML subtype were associated with race. Moreover, the FAB type of t(8; 21) was nearly M2 and mostly of inv (16) were M4, which was consistent with previous reports [5, 6]. Subsequently, much higher initial WBC were observed in childhood patients with inv(16) compared with those with t(8; 21), we suggested that inv(16) was associated with leukocytosis and extramedullary infiltrative manifestations [1]. Interestingly, the percentage of chloroma was lower in patients with inv(16) than those with t(8; 21), and this was never been reported before. In accordance with previous studies, when referred to relapse, BM relapse and CNS relapse were more frequent in patients with inv(16) than those with t(8; 21) [7–9].

Using the large TARGET database of pediatric cases of CBF-AML, we characterized the secondary cytogenetic abnormalities in patients with CBF-AML, defined by either inv(16) or t(8;21). Most previous studies showed that del(9q) and loss of a sex chromosome were more frequent in patients with t(8;21) [10–12]. Similar results were also observed in our study, del(9q), minus X and minus Y had a much higher percentage in t(8;21). Von Neuhoff et al. [4] showed that the 5-year EFS of children with t(8;21) combined with loss of a sex chromosome was significantly higher than that of children with t(8;21) (100% vs 71%, *P* = 0041). Other findings also supported the association of loss of a sex chromosome with a better prognosis in children with t(8;21) [3, 29, 30]. However, Duployez et al. [13] showed that loss of a sex chromosome had no impact on the prognosis of CBF-AML patients in a large mixed cohort study of 73 children and 125 adult patients. In contrast to a previous report by Duployez et al., we found loss of a sex chromosome was not a prognostic factor in our cohort. Klein et al. [2] showed that CBF-AML patients in the del(9q) group (*n* = 104) had a lower CR rate than those in the non-del(9q) group (*n* = 734) (*P* = 0.01), while another study [4] showed a good prognosis for children with the t(8;21) AML subgroup with del(9q). In the present study, patients with del(9q) did not show any significant differences in terms of survival and remission rates. This might be explained by the inconsistent sample size of these studies.

Children with CBF-AML respond well to chemotherapy, and other research centers have reported CR rates of up to 90% after chemotherapy [14–16]. In our cohort, the CR rates were 95.2% in the CBF-AML patients, and 94% and 95.2% in inv(16) and t(8;21), respectively [17, 18]. Although the previously reported mediocre OS and high relapse rate were confirmed in this cohort, the 10-year OS of 84.4% among the CBF-AML patients were relatively good, especially given the number of patients who were diagnosed many years ago. In 2015, AML–Berlin-Frankfurt-Münster (BFM)-98 Study showed the favorable outcome in the subgroups of patients with inv(16) and t(8;21), with an 5-years OS of 87 and 91%, and the 5-years CIR were 16% and 12%, respectively [4]. In the current study, we also demonstrated an excellent 10-years OS of 89.5 and 80.7% but with a relative higher 10-years CIR of 35.2% and 25.4% in the patients with inv(16) and t(8;21), respectively. Compared to our study, the CIR were much lower in the BFM-98 Study, we suggested that the reason was that sample of CBF-AML patients in the BFM-98 Study were smaller (only 99 cases) and the median follow-up were shorter than ours. In spite of these difference, we found patients with inv(16) had a significant higher survival rate and relapse rate than those with t(8;21) in our study. In terms of clinical characters and prognosis, we might concluded that the patients with inv(16) and those with t(8;21) were the two clinically distinct entities.

The c-kit mutation were the most common in CBF-AML children [19, 20]. Recent reports have demonstrated that the prevalence of c-kit mutation in children with CBF-AML was 10-54.5% [8, 19–23]. Chen et al. [21] showed that the incidence of c-kit mutation in children with t(8;21) ranged from 17 to 42%, and 21 to 55% in those with inv(16). In the present study, c-kit mutations were 25.4% among CBF-AML children, and the percentage of c-kit mutation in children with t(8;21) were 24.4%, and 26.9% in those with inv(16). The results of this study were generally consistent with previous findings. The c-kit mutations were widely reported in adults with CBF-AML, and most investigators believed that the mutations suggested a poor prognosis. Tokumasu et al. [23] showed that 46 pediatric patients with t(8;21) accompanying c-kit mutations had a significantly lower EFS than 61 cases without mutations (*n* = 61). Our multivariate analysis also addressed that c-kit mutations were the independent adverse factor that influenced CIR and OS. However, the studies on c-kit mutations in children with CBF-AML were still rare, and the relationship between mutations and prognosis remained controversial. An international, multicenter survey of 97 patients of CBF-AML showed that CBF-AML patients with FLT3-ITD had much lower 4-year relapse-free survival rate compared to the patients without FLT3-ITD (38% vs 80%, *P* = 0.02) [24]. In our study, FLT3-ITD positive demonstrate a poor outcome in terms of OS an CIR, and this was further confirmed in our multivariate analysis. Based on the second strike doctrine, we implied that c-kit mutation and FLT3-ITD mutations play an important role in the pathogenesis of CBF-AML.

The erythromycin plus cytarabine induction chemotherapy regimen and the high-dose eytarabine based consolidation chemotherapy regimen are the clinical standard first-line chemotherapy regimens for CBF-AML [25, 26]. A Cancer and Leukemia Group B Study showed that patients in the two consolidation groups, multicourse HDAC (*n* = 149) and single-course HDAC (*n* = 48), had significant difference on 10-years CIR (41% vs 64%, *P* = 0.009) [27]. CALGB 8461 study demonstrated that the CIR was significantly decreased in patients assigned to receive three to four cycles of HDAC(*n* = 28) compared with patients assigned to one course (*n* = 20) (5-year CIR, 43% v 70%, *P* = 0.03) [28]. In contrast to the two reported results, our study demonstrated that no significant difference were found between four (21.6 g/m^2^ cytarabine) and five (45.6 g/m^2^ cytarabine) chemotherapy courses in terms of CIR and OS. Most interestingly, subgroup analysis showed that CIR of patients with t(8;21) can be decrease by five chemotherapy courses, and this suggested that maybe only patients with t(8;21) could benefit from more cumulative cytarabine exposure.

The impact of GO treatment on outcome was subsequently evaluated in our study. A meta-analysis that included five randomized controlled trials showed that GO treatment improved the risk of relapse and 5-year OS in CBF-AML patients, with a definite survival advantage for CBF-AML patients with GO treatment compared to those without GO treatment(OR = 0.47, 95%*CI*:0.31–0.73, *P* < 0.001) [29–33]. Although our data did not show significant difference between GO and No-GO treatment, the subgroup analysis showed that the patients with inv(16) who did not receive GO had significant higher CIR and similar OS when compared to those with t(8;21). In contrast, inv(16) and t(8;21) receiving GO treatment had comparable outcomes as well as OS and CIR, and this suggested the GO added to conventional chemotherapy improved outcomes for only inv(16). In the current study, we confirmed that the outcome of patients with c-kit mutations could be improved by GO treatment. Thus, we implied that due to c-kit mutations were more common in inv(16), so the patients with inv(16) were also improved by GO treatment.

In summary, we concluded that patients with inv(16) and t(8;21) pediatric AML constitute two separate entities clinically, in that they differ with regard to clinical characteristics, prognosis and treatments. Notably, we showed the impact of GO treatment on patients with inv(16)) and cumulative cytarabine exposure on patients with t(8;21). Furthermore, due to our data, based on a prolonged follow-up, show that the rates of relapse are still disappointing for both patients with inv(16) AML and those with t(8;21) AML, it is important that future studies identify and target therapeutically the leukemogenic mechanisms accountable for molecular and clinical differences between the two cytogenetic groups of CBF AML.

## Supplementary Information

**Additional file 1:** **Supplementary Table 1.** AAML1031 therapeutic regimen in LR patients. **Supplementary Table 2.** AAML0531 therapeutic regimen in LR patients.

## Data Availability

The data sets used and/or analysed during the current study are available from the.corresponding author on reasonable request.
