# Supplementary material for: Outcome and prognostic factors of CBF pediatric AML patients with t(8;21) differ from patients with inv(16)
Source: BMC Cancer. 2023 May 25;23:476. doi: 10.1186/s12885-023-10965-5 (PMC10210276; doi:10.1186/s12885-023-10965-5)
Supplement: Supplementary file 1 — Additional file 1: Supplementary Table 1. AAML1031 therapeutic regimen in LR patients. Supplementary Table 2. AAML0531 therapeutic regimen in LR patients. [file 12885_2023_10965_MOESM1_ESM.docx]

**Supplementary** **Tables**

**Supplementary** **Table** **1** **AAML1031** **Therapeutic** **Regimen In LR Patients**

| **Course and drugs** | **Does** | **Days** |
| --- | --- | --- |
| **Induction Course I** |  |  |
| Cytarabine | 100 mg/m2/dose twice per day IV | 1- 10 |
| Daunomycin | 50 mg/m2/dose IV | 1, 3, 5 |
| Etoposide | 100 mg/m2/dose IV | 1-5 |
| **Induction Course II** |  |  |
| Cytarabine | 1000 mg/m2/dose twice per day IV | 1-4 |
| Mitoxantrone  **Intensification course I** | 12mg/m2/dose IV | 3-6 |
| Cytarabine | 1,000 mg/m2/dose twice per day IV | 1-5 |
| Etoposide  **Intensification course II** | 150 mg/m2/dose twice per day IV | 1-5 |
| Cytarabine | 3,000 mg/m2/dose twice per day IV | 1,2,8,9 |
| Escherichia coli L-asparaginase | 6,000 mg/m2/dose IM | 2, 9 |

**Abbreviations:** IM, intramuscular; IV, intravenous

**Supplementary** **Table** **2** **AAML0531** **Therapeutic** **Regimen in LR Patients**

| Course and drugs | Does | Days |
| --- | --- | --- |
| **Induction Course I** |  |  |
| Cytarabine | 100 mg/m2/dose twice per day IV | 1- 10 |
| Daunomycin | 50 mg/m2/dose IV | 1, 3, 5 |
| Etoposide | 100 mg/m2/dose IV | 1-5 |
| **Induction Course II** |  |  |
| Cytarabine | 100 mg/m2/dose twice per day IV | 1-8 |
| Daunomycin | 50 mg/m2/dose IV | 1, 3, 5 |
| Etoposide | 100 mg/m2/dose IV | 1-5 |
| **Intensification course I** |  |  |
| Cytarabine | 1,000 mg/m2/dose twice per day IV | 1-5 |
| Etoposide  **Intensification course II** | 150 mg/m2/dose IV | 1-5 |
| Mitoxantrone | 12mg/m2/dose IV | 3-6 |
| Cytarabine | 1,000 mg/m2/dose twice per day IV | 1-4 |
| **Intensification course III** |  |  |
| Cytarabine | 3,000 mg/m2/dose twice per day IV | 1, 2, 8, 9 |
| Escherichia coli L-asparaginase | 6,000 U/m2/dose IM | 2, 9 |

**Abbreviations:** IM, intramuscular; IV, intraveno
